# Correlation between blood concentration of roxadustat and clinical efficacy in patients with anemia of chronic kidney disease

**DOI:** 10.1097/MD.0000000000033564

**Published:** 2023-04-14

**Authors:** Yanjing Zhang, Yu Jing, Chunhua Zhou

**Affiliations:** a Department of Clinical Pharmacy, The First Hospital of Hebei Medical University, Shijiazhuang, Hebei, China.

**Keywords:** blood concentration, clinical efficacy, correlation, renal anemia, roxadustat

## Abstract

Roxadustat has been associated with the efficacy and safety in patients with chronic kidney disease-related anemia. However, the relationship between roxadustat blood concentration and clinical efficacy is lacking. To explore of the correlation between clinical efficacy and blood concentration of roxadustat in patients with renal anemia of chronic kidney diseases, so as to provide reference for rational clinical drug use. A total of 46 patients were selected with a diagnosis of renal anemia who were prescribed roxadustat at the department of nephrology of the First Hospital of Hebei Medical University from December 2019 to March 2020. The roxadustat blood concentration was determined at 12 weeks of treatment, according to the cumulative response rate, patients were divided into the response group and the nonresponse group, and the relationship between roxadustat blood concentration and treatment effect was analyzed. We also explored the correlation between various factors and the blood concentration. The patients in the response group had higher roxadustat blood concentrations than the nonresponse group (*P* < .05), and there was no correlation between blood concentration and clinical characteristics such as age, gender, and dosage (*P* > .05). The blood concentration of roxadustat was correlated with clinical efficacy. The higher the blood concentration, the better the clinical efficacy, meaning it might be a predictor of efficacy.

## 1. Introduction

As a serious public health challenge with increasing global mortality and morbidity, chronic kidney disease (CKD) is characterized by progressive loss of kidney function, leading to dialysis or kidney transplantation.^[1]^ The overall prevalence of CKD in China is 10.8% and is expected to increase in the coming decades, particularly in rural areas.^[2]^ Anemia is one of the most severe and inevitable complications associated with CKD.^[3]^ Currently, the standard of care includes oral or intravenous iron supplementation, erythropoiesis-stimulating agents, and red blood cell transfusion.^[4]^ Multiple trials have demonstrated that these anti-anemia therapies increased the potential risks (thrombosis of arteriovenous fistula, hypertension, stroke).^[5,6]^ Therefore, new strategies are required to address these problems.

Roxadustat is an orally administered, small molecule hypoxia-inducible factor (HIF) prolyl hydroxylase inhibitor for the treatment of anemia in patients with CKD. The drug inhibits HIF prolyl hydroxylase enzymes and promotes erythropoiesis by increasing endogenous erythropoietin, improving iron regulation, and reducing hepcidin.^[7]^ The apparent clearance of roxadustat is 1.2 to 2.65 L/hours, and the apparent volume of distribution is 22 to 57 L, the elimination half-life is 9.6 to 16 hours, the plasma binding is 99% and The pharmacokinetic profile of roxadustat was unaffected by hemodialysis/hemodiafiltration.^[8,9]^The effects of roxadustat on hemoglobin (Hb) are linear, long-lasting, and likely irreversible, and the concentration at the half-maximum effect was 10 to 36 µg/mL.^[10]^ Numerous studies have shown that roxadustat is safe and effective in patients with CKD,^[11]^but little literature has been reported on the blood concentration of roxadustat. The main aim of this study was to analyze the relationship between blood concentrations and curative effect. The secondary aim was to explore the factors of affecting drug concentration.

## 2. Materials and methods

### 2.1. General data

This is a retrospective observational study. From December 2019 to March 2020，outpatients in First Hospital of Hebei Medical University diagnosed with CKD were treated with roxadustat. The study was approved by the Ethics Committee of the First Hospital of Hebei Medical University (20220753).

Inclusion criteria were as follows: Age 18 to 90 years; End-stage kidney disease patients who are receiving maintenance hemodialysis; Renal anemia, defined as Hb < 120 g/L or Hb is maintained normal by ESA; Willing to participate in the study with treatment of roxadustat and all selected patients who had been treated with recombinant human EPO were administered with roxadustat to correct anemia 1 week after discontinuation of EPO 10,000 IU per week and 3 days after discontinuation of EPO 3000 IU per week^[12]^; Exclusion criteria were as follows: Patients who cannot complete the follow-up study; Patients with poor control of blood pressure; Patients with history of malignancy; Patients with severe liver impairment or active hepatitis; Patients with hereditary hematologic diseases such as thalassemia, sickle cell anemia, or other known causes of anemia other than CKD.

### 2.2. Methods

#### 2.2.1. Treatment plan

The dosage of roxadustat was 100 mg for patients weighing 45 to 60 kg and 120 mg for patients weighing > 60 kg. The drug was taken orally 3 times a week. The dosage of roxadustat was adjusted according to the degree of Hb change over the past 4 weeks, as described in the drug’s instructions.

#### 2.2.2. Blood concentration detection

The venous blood samples about 2 to 3 mL were collected before the oral administration of roxadustat in heparin tube, and plasma samples were obtained by centrifuging the blood samples at 4000 r/minutes for 10 minutes, and the supernatants were stored at − 80°C until required for analysis. Plasma concentrations of roxadustat were measured using liquid chromatography–tandem mass spectrometry method. Physcion was used as the internal standard. The gradient elution was performed on a Phenomenex Kinetex C_18_ column (3.0 mm × 100.0 mm, 2.6μm) at a flow rate of 0.50 mL/minutes with mobile phase methanol (0.1% formic acid)–water (0.1% formic acid, 10 mmol/L ammonium acetate) solution. The injection volume was 5 μL. Electron spray ionization ion source was used for mass spectrometry and positive ion multiple reaction monitoring scanning detection. The method was proved to be liner in the range of 1 to 1000 ng/mL for roxadustat. The limit of quantification for roxadustat was 1 ng/mL. The intra–day and inter–day RSD of roxadustat were <10%. Extraction recovery rate was 87.96% to 102.82%.

#### 2.2.3. Evaluation of clinical efficacy

According to the cumulative response at the time of blood concentration determination, patients were divided into the response group and the nonresponse group.^[13]^ Response group: the Hb level ≥ 100 g/L or change in Hb from baseline ≥ 10g/L, Nonresponse group: the Hb level < 100 g/L and change in Hb from baseline ≥ 10g/L.

### 2.3. Statistical analysis

Data were analyzed using SPSS version 26.0. *P* < .05 was considered statistically significant. Continuous variables are expressed as the mean ± standard deviation, and classified variables are described as numbers and percentages. When the data fit the normal distribution, an independent 2-sample *t* test was used in the comparison of means between groups. When the data did not conform to a normal distribution, the Mann–Whitney *U* test was used for unpaired continuous variables between groups. General linear bivariate spearman linear correlation was used to test the relationship between roxadustat concentration and related characteristics.

## 3. Results

### 3.1. The distribution of blood concentration of roxadustat

A total of 46 CKD patients were tested for blood drug concentration after roxadustat treatment, the average blood concentration of roxadustat was 3327.96 ± 4454.04 ng/mL (range of 109 to 16,800 ng/mL).

### 3.2. Clinical efficacy

Hb was 102.22 ± 15.33 g/L in the pretreatment phase and 109.93 ± 16.37 g/L in the posttreatment phase, and the serum iron was 12.59 ± 4.39 μmol/L in the pretreatment phase and 17.52 ± 6.82 μmol/L in the posttreatment phase, which suggested that roxadustat increased Hb and iron, and the changes were statistically significant (*P* < .05). The effects of roxadustat on others clinical characteristics are shown in Table 1.

**Table 1:** The effects of roxadustat on clinical characteristics.

Parameter	Reference value	Pretreatment(x ± s)	Posttreatment(x ± s)	*P* value
Hb (g/L)	115–150	102.22 ± 15.33	109.93 ± 16.37	<.05
Serum creatinine (μmol/L)	57–111	872.06 ± 271.63	902.72 ± 302.33	.192
Uric acid (μmol/L)	208–428	409.23 ± 77.47	415.69 ± 85.07	.443
BUN (mmol/L)	3.6–9.5	23.77 ± 6.49	27.13 ± 7.83	<.01
K^+^ (mmol/L)	3.5–5.3	4.84 ± 0.81	4.92 ± 0.77	.592
Ca^+^ (mmol/L)	2.2–2.6	2.13 ± 0.22	2.13 ± 0.22	.869
P (mmol/L)	0.8–1.6	1.97 ± 0.68	2.06 ± 0.62	.382
Ferritin (μg/L)	11–306	109.82 ± 94.67	137.51 ± 170.89	.075
Iron (μmol/L)	7.7–30	12.59 ± 4.39	17.52 ± 6.82	<.05
TIBC (μmol/L)	45–75	43.76 ± 10.52	54.21 ± 61.08	.418
UIBC (μmol/L)	31–51	31.17 ± 11.91	30.01 ± 30.56	.872
β_2_MG (mg/L)	1–3	33.99 ± 8.08	32.53 ± 8.51	.500
PTH (ng/L)	230–630	472.75 ± 598.55	404.22 ± 367.87	.302
CRP (mg/L)	0–3	2.27 ± 3.53	7.06 ± 13.52	.081
Albumin (g/L)	40–55	38.29 ± 3.53	36.86 ± 4.42	.111

Hb = hemoglobin.

### 3.3. Relationship between roxadustat concentration and clinical efficacy

A total of 37 patients were included in the response group, accounting for 80.43% (37/46); 9 patients, accounting for 19.57% (9/46), were included in the nonresponse group. The blood concentrations of roxadustat in the nonresponse group were lower than those in the response group, and the differences were statistically significant (*P* < .05) (Table 2).

**Table 2:** Comparison of baseline data and main laboratory indicators between the 2 groups.

Characteristics	Response group	Nonresponse group	*P* value
Number of patients	37	9	–
Gender			
Male	23	7	.463
Female	14	2	
Age (yr)	53.14 ± 2.14	50.67 ± 3.80	.576
BMI (kg/m^2^)	22.33 ± 0.62	25.04 ± 1.94	.089
Height (cm)	168.00 ± 1.43	171.56 ± 2.83	.271
Weight (kg)	63.45 ± 2.36	73.22 ± 5.08	.107
Dosage (mg)	105.00 ± 2.13	126.67 ± 4.41	<.01
Hb (g/L)	115.86 ± 12.52	92.44 ± 11.71	<.01
Serum creatinine (μmol/L)	902.50 ± 305.41	851.39 ± 210.40	.638
Uric acid (μmol/L)	411.58 ± 91.45	403.77 ± 79.85	.967
BUN (mmol/L)	26.56 ± 7.65	27.41 ± 9.36	.776
K + (mmol/L)	4.92 ± 0.78	4.90 ± 0.69	.965
Ca + (mmol/L)	2.15 ± 0.19	2.21 ± 0.26	.394
P (mmol/L)	2.08 ± 0.58	1.65 ± 0.29	<.05
Ferritin (μg/L)	119.87 ± 166.12	204.67 ± 201.53	.198
Iron (μmol/L)	16.70 ± 4.37	22.93 ± 9.36	<.05
TIBC (μmol/L)	40.00 ± 15.47	85.71 ± 106.47	.145
UIBC (μmol/L)	30.14 ± 33.31	21.36 ± 10.05	.935
β2MG (mg/L)	32.84 ± 8.50	26.58 ± 7.56	.19
PTH (ng/L)	441.16 ± 433.39	349.20 ± 219.23	.569
CRP (mg/L)	8.48 ± 16.35	3.82 ± 5.01	.64
Albumin (g/L)	36.83 ± 4.82	36.43 ± 4.33	.414
Plasma concentration(ng/mL)	3914.00 ± 4785.58	918.67 ± 643.78	<.05

Hb = hemoglobin.

### 3.4. Correlations of clinical characteristics and plasma concentrations of roxadustat

Potential clinical characteristics that may have influenced the concentration of roxadustat were summarized and calculated in Table 3. No correlation between the plasma concentration of roxadustat and other clinical characteristics was found (*P* > .05).

**Table 3:** Correlations of clinical characteristics and plasma concentrations of roxadustat.

Number	Spearson *r*	*P* value
Gender	−0.143	.344
Age (yr)	0.034	.825
BMI (kg/m^2^)	−0.013	.931
Height (cm)	0.356	.056
Weight (kg)	0.136	.372
Dosage (mg)	0.010	.948
Hb (g/L)	−0.024	.877
Ferritin (μg/L)	−0.046	.819
Iron (μmol/L)	−0.105	.602
TIBC (μmol/L)	−0.172	.390
UIBC (μmol/L)	−0.149	.458

Hb = hemoglobin.

## 4. Discussion

Anemia is a common complication of CKD caused by impaired oxygen sensing during renal failure.^[14]^ As a HIF prolyl hydroxylase inhibitor, the novel agent roxadustat may increase endogenous erythropoietin concentrations by regulating the HIF, which is activated via the inhibition of certain activated domains.^[15]^ In our study, roxadustat demonstrated prospective efficacy for the treatment of CKD patients with anemia, the Hb level after roxadustat treatment was significantly higher than the baseline value, and the iron metabolism-related indicators were significantly improved, which is consistent with previous studies.^[16,17]^

Although many studies have verified the effectiveness of roxadustat for renal anemia,^[18,19]^ the optimal duration of treatment and dosage have not been well defined so far. As far as we know, no data are available for determining the concentration-effect relationship of roxadustat. All patients who were enrolled had been exposed to roxadustat for at least 12 weeks and were stable at the current dosage for 1 weeks to ensure a steady blood concentration. Our data showed a significant correlation between clinical efficacy and plasma concentration. The plasma concentration of roxadustat in the response group was higher than that in the nonresponse group. Statistical analysis showed that the difference of roxadustat concentration between the 2 groups was statistically significant (*P* < .05). Some factors, such as Hb, iron metabolism, residual renal function and dialysis adequacy have been demonstrated as predictors of the response to roxadustat.^[20]^ As shown in our data, the plasma concentration of roxadustat also might be a potential reference for determining the response.

The researchers developed a population pharmacokinetic model of roxadustat in the target population, The results showed that none of the investigated intrinsic or extrinsic factors (such as sex, bodyweight, dialysis status, albumin, and dose) resulted in a significant change in roxadustat exposure outside of the defined no-effect boundaries.^[21]^ Consistent with the findings, the results of the bivariate Spearman linear correlation test showed that gender, age and other factors had no clinically relevant effect on the blood concentration of roxadustat. It is according to the pharmacology of reasoning, and the real reason further analysis is needed.^[10]^

Our study has the following limitations. First, this was a single-center study with a relatively small sample size, which might lead to the uneven distribution of the ratio in our cohort. Second, this was an observational study and selection bias could not be completely eliminated. Therefore, the correlations of clinical efficacy and plasma concentrations of roxadustat in CKD patients presented here should be regarded as preliminary. Further prospective, larger-scale clinical trials are needed to verify the finding.

## 5. Conclusion

In conclusion, there is a correlation between roxadustat concentration and the efficacy. The blood concentration of roxadustat in the response group was higher than the nonresponse group for CKD patients treated with roxadustat, which might be helpful for the prediction of efficacy.

## Acknowledgments

The authors are thankful for clinicians and nurses in the department of nephrology, the First Hospital of Hebei Medical University, Shijiazhuang, Hebei, China.

## Author contributions

**Conceptualization:** Chunhua Zhou.

**Data curation:** Chunhua Zhou.

**Formal analysis:** Chunhua Zhou.

**Funding acquisition:** Chunhua Zhou.

**Investigation:** Chunhua Zhou.

**Methodology:** Yanjing Zhang, Chunhua Zhou.

**Project administration:** Yanjing Zhang, Chunhua Zhou.

**Resources:** Yanjing Zhang.

**Software:** Yanjing Zhang.

**Supervision:** Yanjing Zhang.

**Validation:** Yanjing Zhang, Yu Jing.

**Visualization:** Yanjing Zhang, Yu Jing.

**Writing – original draft:** Yanjing Zhang, Yu Jing.

**Writing – review & editing:** Yu Jing.
